# Efficacy of 100-U Onabotulinumtoxin A Treatment in Female Idiopathic Overactive Bladder—A Prospective Follow-Up Study

**DOI:** 10.1007/s00192-025-06047-8

**Published:** 2025-01-31

**Authors:** Mona Nurkkala, Heini Salo, Terhi Piltonen, Henri Sova, Henna-Riikka Rossi

**Affiliations:** Department of Obstetrics and Gynaecology, Medical Research Center Oulu, Research Unit of Clinical Medicine, University of Oulu and Oulu University Hospital, Kajaanintie 50, BOX 5000, 90014 Oulu, Finland

**Keywords:** Female idiopathic overactive bladder, Onabotulinumtoxin A

## Abstract

**Introduction and Hypothesis:**

Overactive bladder (OAB) affects 11–17% of the female population. First-line treatment with lifestyle modifications and second-line therapy with medications are often limited by inadequate efficacy or pharmacological side effects. This study was aimed at assessing the effect of 100 U onabotulinumtoxin A treatment on idiopathic OAB (iOAB) as a second-line treatment.

**Methods:**

This prospective follow-up study involved 94 women who received onabotulinumtoxin A treatment at a dose of 100 U as a second-line treatment for iOAB at the Department of Obstetrics and Gynecology, Oulu University Hospital, Finland, between May 2018 and December 2023. The impact of the treatment on iOAB symptoms was evaluated 3 months after administration using self-reported symptoms and the following internationally validated questionnaires: Visual Analogue Scale (VAS), Incontinence Impact Questionnaire (IIQ-7), and Urogenital Distress Inventory (UDI-6). Postoperative complications were assessed.

**Results:**

The follow-up data were obtained from 74 (78%) patients, of whom 66 (95.7%) reported a good outcome and 3 (4.3%) reported a poor outcome. Incontinence episodes, the number of incontinence pads needed, and daily micturitions were significantly reduced. For all questionnaires, the total scores decreased significantly after the treatment (VAS 8.27 ± 1.78 vs 3.50 ± 3.08, *p* < 0.001; IIQ-7 72.14 ± 20.55 vs 28.73 ± 29.40, *p* < 0.001; UDI-6 55.01 ± 18.86 vs 29.66 ± 22.03, *p* < 0.001). Postoperative urinary tract infection occurred in 9 patients (9.6%), whereas urinary retention occurred in 6 patients (6.4%).

**Conclusions:**

Onabotulinumtoxin A (100 U) demonstrates good effectiveness in the second-line treatment of female iOAB.

## Introduction

Overactive bladder (OAB) is defined as urinary urgency, with or without urge incontinence, usually accompanied by increased frequency and nocturia, in the absence of urinary tract infection (UTI) or other obvious pathology [1–3]. OAB is classified as idiopathic (iOAB) if there is no specific condition or pathophysiology, usually neurological, causing the symptoms [1]. OAB affects 11–17% of the female population worldwide, with the prevalence increasing with age [4, 5]. OAB has been shown to diminish women’s quality of life, adversely impacting self-confidence, sexual function, mental health, work ability, and social and professional relationships [6–9].

The first-line treatment of OAB includes patient education, behavioral therapies, and the administration of antimuscarinic or beta-3 agonist agents, and these should always be tested before considering second-line treatments [6, 10]. However, sometimes the insufficient effectiveness of these treatments and the many side effects of the medications, including dry mouth, cognitive problems, constipation, elevated blood pressure, and heart palpitations, limit their long-term use [11, 12]. Furthermore, OAB medication requires constant, lifelong use, resulting in substantial costs for both patients and society [13, 14].

Several studies have shown that onabotulinumtoxin A treatment reduces urinary frequency and urgency, urge incontinence, and nocturia, and improves quality of life [4, 8, 15], and it has been approved as a second-line treatment for drug-resistant iOAB by several national and international guidelines [6, 10, 11, 16–19]. Risks related to onabotulinumtoxin A treatment include UTI (22–35%), urinary retention (8–42%), and the possible need for temporary clean intermittent catheterization (CIC) [9, 12, 20], which are both known to be dose dependent [8, 11]. Previous studies of onabotulinumtoxin A in the treatment of OAB have been conducted with varying patient selection and outcome measures, with onabotulinumtoxin A being administered at doses ranging from 50 to 300 U [11, 12]. Moreover, many studies have included both men and women, as well as both neurogenic and idiopathic OAB [6, 12, 15]. Comparatively few studies have focused solely on women with idiopathic OAB with a 100-U dose of onabotulinumtoxin A [1, 21].

Onabotulinumtoxin A treatment at a dose of 100 U has been performed at the Department of Obstetrics and Gynecology, Oulu University Hospital, since 2018 as a second-line treatment for women suffering from iOAB. The aim of the present study was to assess the effect of 100 U onabotulinumtoxin A as a second-line treatment of iOAB symptoms among female patients via internationally validated and free questionnaires. Second, the rate of adverse effects (AEs) and the elapsed time between treatments were assessed.

## Materials and Methods

The materials for the study were collected from the outpatient clinic of the Department of Obstetrics and Gynecology at Oulu University Hospital. This prospective follow-up study involved 94 women suffering from iOAB for whom conservative treatment, including lifestyle modifications and medications, were inadequate/inappropriate, and onabotulinumtoxin A was planned as a second-line treatment for iOAB according to the hospital’s care practice. All the patients were informed about the study by a urogynecologist when they came to the clinic. Furthermore, the participants were given the questionnaires to be answered before the operation, and they gave their consent to participate in the study by filling out and returning the written questionnaires. They also had the right to withdraw from the study at any point without any impact on treatment procedure. Women with iOAB were identified by clinical interviews, voiding diaries that reported increased micturition frequency (more than 8 times per day) and small urine volumes with normal fluid intake, validated UI questionnaires (Detrusor Instability Score [DIS], Urinary Incontinence Severity Score [UISS], and Overactive Bladder Symptom Score [OABSS]), and a gynecological examination, including pelvic ultrasound scans [22, 23]. Women with obvious neurogenic OAB (spinal cord injury, etc.) were sent to the urological department and thus excluded from the study population. Furthermore, women who were incapable of CIC were excluded owing to possible retention. All injections were performed at the Department of Obstetrics and Gynecology at Oulu University Hospital between May 2018 and December 2023. Ciprofloxacin 500 mg, administered perorally, was used as an antimicrobial prophylaxis on the treatment day. A total of 100 U of onabotulinumtoxin A was administered via 20 intradetrusor injections to the bladder under local anesthesia using lidocaine hydrochloride gel and monitoring by a rigid cystoscope. All injections were performed by two specialized urogynecologists (H-RR, HS). Postoperative urination was confirmed before the patients were discharged. After the treatment, the patients were advised to contact the hospital in the event of any AEs, especially difficulty urinating, symptoms of infection, or worsening of urge symptoms. For those patients who did contact the hospital, a urine sample was taken and examined to rule out infections, and the residual was measured with an ultrasound scan. Possible infections were treated with an adequate antibiotic and, if the residual was more than 200 ml, the patient was instructed to carry out CIC. All postoperative contacts were collected and documented from the patient register of Oulu University Hospital, and these were classified as UTI, urinary retention, pain, and constant urine leakage. Additionally, all retreatments and the elapsed time between the first and second treatment were documented. Information about complications and the elapsed time until retreatment was collected from the beginning of the study until the end of March 2024.

The life impact and symptom distress of OAB were assessed using the Incontinence Impact Questionnaire short form (IIQ-7) and Urogenital Distress Inventory short form (UDI-6) [24]. Disadvantage to everyday life was measured on the Visual Analogue Scale (VAS) with the question “How much disadvantage does urinary incontinence cause you?” with answers ranging from 0 (none) to 10 (very much) [22]. These three internationally standardized and validated questionnaires were administered preoperatively and were repeated 3 months after treatment, after which the scores were compared [8, 15]. Furthermore, self-reported daily micturition frequency, incontinence episodes, and incontinence pad use were documented preoperatively and at the 3-month follow-up according to the known duration of effectiveness of onabotulinumtoxin A. The Patient Global Index of Improvement (PGI-I) questionnaire, which has a transition scale (from 1 to 7), was used to assess the effect of 100 U onabotulinumtoxin A treatment on women’s postoperative well-being. The effect was dichotomized into good (PGI-I 1–4) and poor (PGI-I 5–7) outcomes, the characteristics of which were compared [25]. Finally, the results in these subgroups among women under and over the mean age of the study population, as well as between women with overweight and those of a normal weight, were compared.

Several patient-related covariates, such as age and body mass index (BMI) at the time of treatment, parity, and smoking status, were collected from the patient register of Oulu University Hospital. Information about neurological diseases was collected from the patient register using International Classification of Disease (ICD-10) codes (diseases of the nervous system G00–G99), whereas information about prior hysterectomies or pelvic organ prolapse operations was retrieved via NOMESCO Classification of Surgical Procedure (NCSP) codes (vaginal hysterectomy = LCD10, LCD40, LEF13, and LEF14; laparoscopic hysterectomy = LCD01, LCD04, LCD11, LCD31, and LCD97; abdominal hysterectomy = LCD00, LCD30, and LCD96; colporrhaphy anterior = LEF00; and colporrhaphy posterior = LEF03). Furthermore, data about prior UI operations (LEG10, LEG12, LEG13, LEG96) and urethral bulking agent injection treatments (KDV20 and KDV22) were collected. Last, data about anticholinergic or β3-agonist medication usage as well as the reason for possible discontinuation of these medications were collected from the patient register.

IBM SPSS Statistics version 28 was used for the statistical analysis. Continuous variables were calculated as means with standard deviations, and categorical variables were calculated as frequencies. The differences in continuous variables were analyzed using an independent samples *t* test or a paired samples *t* test, as appropriate, and a Chi-squared test was used to analyze the differences in the categorical parameters. A two-sided *p* value < 0.05 was considered statistically significant.

## Results

The preoperative characteristics of the study population are shown in Table 1, first for “all participants” and then for the “good outcome” and “poor outcome” groups separately. The mean age of the patients was 67.73 ± 11.15 years (*n* = 94, from 28 to 92 years). All patients had previously used a UI medication, but only 40 (42.6%) were current users. More than one-half of the patients (*n* = 54, 57.4%) had discontinued the medication before the treatment, with 42 (44.7%) discontinuing owing to insufficient efficacy and 12 (12.8%) owing to adverse side effects (Table 1).

**Table 1:** Preoperative patient characteristics

Characteristic	All (*n* = 94)	Good outcome (PGI-I 1–4), (*n* = 66, 95.7%)	Poor outcome (PGI 5–7), (*n* = 3, 4.3%)	*p* value
Age (years), mean ± standard deviation	67.73 ± 11.15	68.41 ± 10.96	77.00 ± 2.65	0.183
Current UI medication, *n* (%)	40 (42.6)	27 (40.9)	0 (0)	0.156
UI medication quit, *n* (%)	54 (57.4)	39 (59.1)	3 (100)	0.156
Insufficient response, *n* (%)	42 (44.7)	30 (45.5)	3 (100)	0.064
Adverse side effect, *n* (%)	12 (12.8)	9 (13.6)	0 (0)	0.493
BMI (kg/m^2^), mean ± standard deviation	29.68 ± 5.28	29.83 ± 5.59	27.90 ± 2.84	0.559
Parity, mean ± standard deviation	2.28 ± 1.34	2.22 ± 1.41	3.00 ± 1.00	0.347
Smoking, *n* (%)	3 (4.8)	3 (7.1)	0 (0)	0.632
Prior hysterectomy, *n* (%)	35 (37.6)	21 (32.3)	1 (33.3)	0.970
Prior POP surgery, *n* (%)	21 (22.8)	16 (25.0)	1 (33.3)	0.746
Prior UI surgery, *n* (%)	31 (33.3)	24 (36.9)	1 (33.3)	0.900
Prior bulking agent treatment, *n* (%)	9 (9.6)	7 (10.6)	1 (33.3)	0.229
Neurological disease, *n* (%)	30 (31.9)	22 (33.3)	3 (100)	0.019

Significance tests for continuous variables were performed by using the independent samples *t* test or the Mann–Whitney *U* test, as appropriate. *p* value < 0.05 was considered significant

Differences in numbers vary in analyses as the result of missing data

*BMI* Body Mass Index, *POP* pelvic organ prolapse, *UI* urinary incontinence

Responses to the postoperative questionnaires were received from 74 (78%) patients. In total, 66 (95.7%) of the respondents reported a good outcome, whereas 3 (4.3%) reported a poor outcome, after onabotulinumtoxin A treatment. No significant differences were found with respect to age, BMI, parity, smoking, or prior hysterectomy or urogynecology operations between the good and poor outcome groups (Table 1). All three patients reporting a poor outcome had a diagnosis of neurological disease compared with 22 women in the good outcome group (100% vs 33.3%, *p* = 0.019). The diagnoses of the three patients reporting a poor outcome were sleep apnea, Parkinson’s disease, and nerve root compression.

**Fig. 1:**
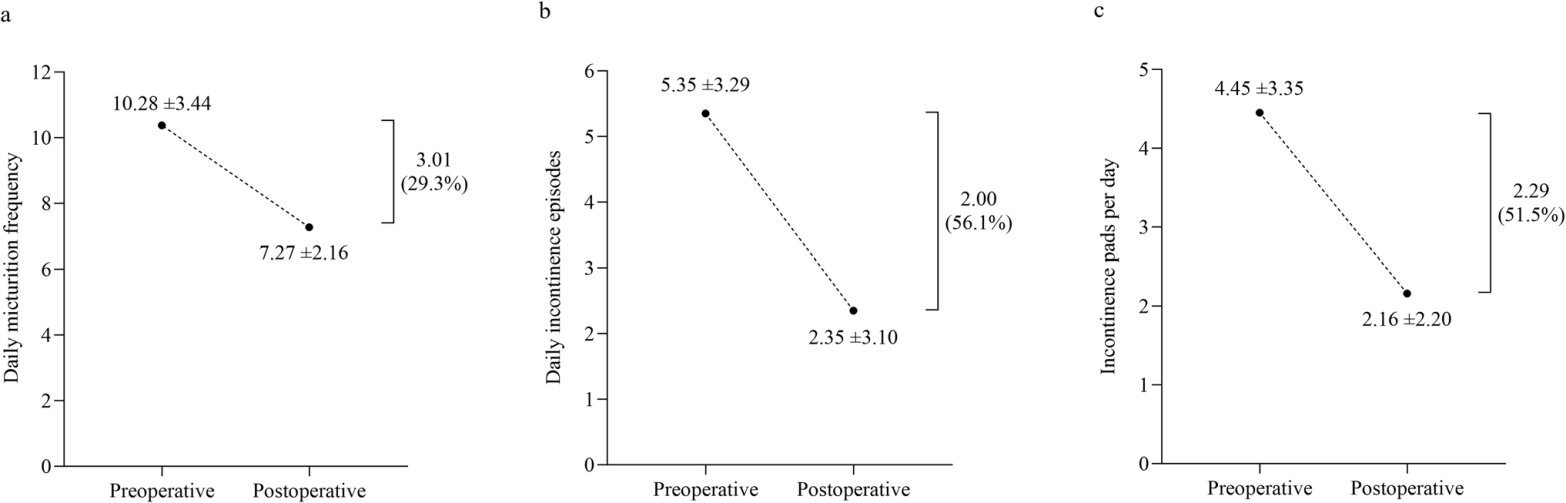
Comparison of **a** daily micturition frequency, **b** daily incontinence episodes, and **c** incontinence pads used per day preoperatively and at 3 months postoperatively

**Fig. 2:**
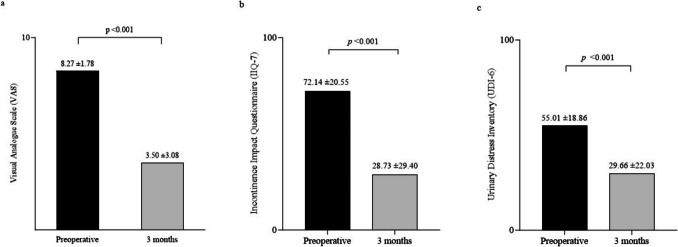
Comparison of **a** Visual Analogue Scale (VAS), **b** Incontinence Impact Questionnaire (IIQ-7), and **c** Urogenital Distress Inventory (UDI-6) preoperatively and at 3 months after the treatment

**Fig. 3:**
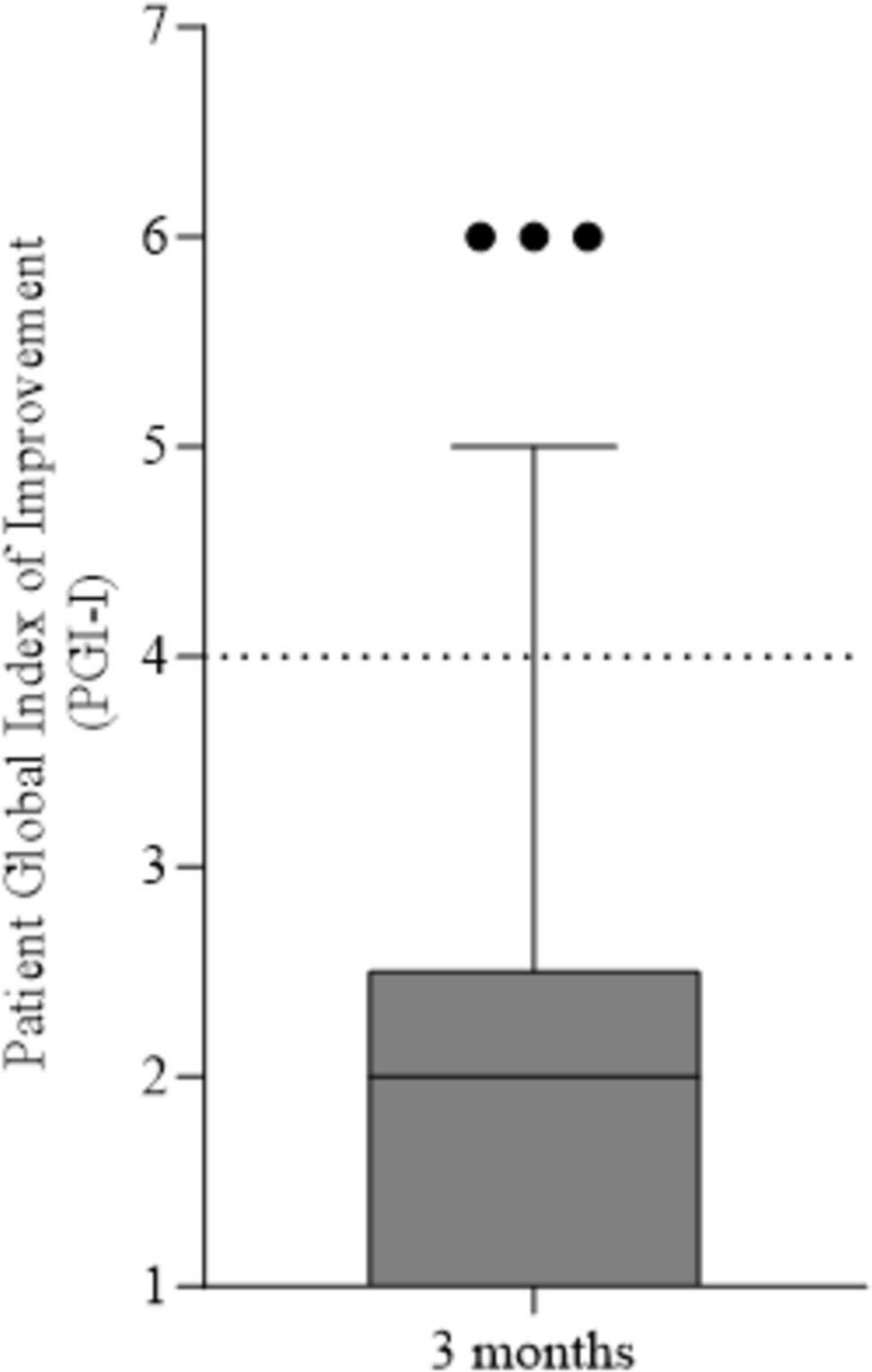
Patient Global Index of Improvement (PGI-I) at 3 months after the treatment. Box-plot whiskers stand for 10th percentile and 90th percentile of the data set

At 3 months after treatment, the number of daily micturitions had decreased by 30% per day (from a mean number of micturitions per day of 10.38 ± 3.44 to 7.27 ± 2.16, *p* < 0.001). Daily incontinence episodes decreased by 56% after treatment compared with preoperatively (from 5.35 ± 3.29 to 2.35 ± 3.10, *p* < 0.001), showing a mean decrease of three episodes per day. Correspondingly, the number of incontinence pads needed per day reduced by 51.5% (from 4.45 ± 3.35 to 2.16 ± 2.20, *p* < 0.001), with a mean decrease of two pads per day (Fig. 1). Compared with the preoperative questionnaire scores, postoperative scores on the VAS, IIQ-7, and UDI-6 decreased significantly during the 3-month follow-up (VAS 8.27 ± 1.78 vs 3.50 ± 3.08, *p* < 0.001; IIQ-7 72.14 ± 20.55 vs 28.73 ± 29.40, *p* < 0.001; UDI-6 55.01 ± 18.86 vs 29.66 ± 22.03, *p* < 0.001; Fig. 2). The postoperative PGI-I score (mean 1.99 ± 1.19) is shown in Fig. 3. The questionnaire results regarding urinary frequency, incontinence episodes, and incontinence pads needed per day were similar when comparing the subgroups of women under and over the mean age (Supplemental Table 1a). Last, the results among overweight women compared with women of normal weight were better: especially the number of incontinence pads needed per day and UDI-6 scores reduced significantly more in women with overweight (Supplemental Table 1b).

Postoperative complications occurred in 16 (17.0%) of the patients. The most common complications were UTI (*p* = 9, 9.6%) and urinary retention with the need for short-term CIC (*p* = 6, 6.4%). Two (2.1%) patients reported constant urine leakage, whereas one (1.1%) reported postoperative pain. No significant differences in preoperative characteristics were found when comparing the retention group with the no retention group (Supplemental Table 2). Of the 94 patients, 43 (45.7%) had received at least one retreatment by the end of the study in March 2024. For patients receiving retreatments within the study period, the mean interval between the first and second treatment was 15 (± 10.46) months, ranging from 4 to 66 months. For preoperative characteristics, smoking was significantly more common in retreated patients than in non-retreated patients (Supplemental Table 3).

## Discussion

In this prospective follow-up study, we showed that 100-U onabotulinumtoxin A treatment is a safe and effective second-line treatment for women suffering from iOAB, even at older age and higher BMI. OAB symptoms were significantly relieved 3 months after the treatment, with 95% of the patients reporting good results according to the validated questionnaires. Furthermore, 100-U onabotulinumtoxin A treatment resulted in an acceptable number of AEs, which occurred in 17% of the patients, mostly UTI or retention. In iOAB patients who received retreatments, the effect of the first 100-U onabotulinumtoxin A treatment lasted on average a year.

As in our study, previous studies have also demonstrated that onabotulinumtoxin A is an effective treatment for OAB and is thus already recommended by several national and international guidelines as a second-line therapy treating in iOAB when conservative treatment is insufficient [6, 10, 11, 16–19, 21, 26]. However, most of these previous studies also included all phenotypes of OAB, including neurogenic [21, 26], and the doses of onabotulinumtoxin A administered in these studies varied from 100 to 300 U [12, 15, 26–28]. In our study, the number of patients who achieved good results was even larger and the effect of the first treatment with onabotulinumtoxin A persisted longer than observed in most of the previous studies [1, 26]. This may be because we only included patients with iOAB. In 2013, two large, randomized, double-blinded, placebo-controlled trials were published on the effectiveness of 100-U onabotulinumtoxin A injections in the treatment of iOAB with UI [15, 27]. Chapple and colleagues included 548 patients [15], whereas Nitti and colleagues included 557 patients [27], of whom around one-half in both studies were treated with a placebo. After treatment with onabotulinumtoxin A, 62.8% of the patients in Chapple et al.’s study [15], and 60.8% of the patients in Nitti et al.’s study [27], reported positive outcomes at the 3-month follow-up based on subjective symptoms, such as UI episodes per day. Meanwhile, in our study, 95.7% of women with iOAB reported positive outcomes on the PGI-I 3 months after the treatment. Contrary to our study, these aforementioned studies included both men and women, whereas we focused only on women. Thus, according to our study, onabotulinumtoxin A treatment with a dose of 100 U demonstrates excellent efficacy, specifically for urogynecological patients with iOAB.

In this study, we used internationally validated questionnaires demonstrating the subjective efficacy of onabotulinumtoxin A treatment. Aside from our study, only that conducted by Gousse et al. used the same questionnaires—namely, the UDI-6 and the VAS—in a follow-up study of 60 patients treated with onabotulinumtoxin A, at doses of 100 U and 150 U [2]. In their study questionnaires were administered before and 6 weeks after the treatment, with the results revealing significant improvement in both treatment arms with no statistically significant difference between the 100-U and 150-U doses. The UDI-6 scores in their study decreased by 59.8%, whereas the VAS scores decreased by 61.3%—in our study, we observed a 46.1% decrease in UDI-6 scores and a 57.7% decrease in VAS scores. However, in the previous study, the percentage of patients who did not benefit from the treatment was not reported [2]. Otherwise, most prior studies have focused on quality-of-life questionnaires, not subjective cure of symptoms for OAB specifically aimed at urogynecology.

To our knowledge, only two women-only studies on 100-U onabotulinumtoxin A treatment have previously been conducted. In the first, a retrospective study of 136 iOAB patients, 66% of the patients exhibited clinical improvement in symptoms and the mean interval of retreatments (17.5 months) [1]. The time elapsed until retreatment in that study was longer than in earlier studies including all phenotypes of OAB, and its findings were in line with those of our study with a mean interval of 15 months. However, as opposed to our study, which was prospective and included internationally validated and free questionnaires about the subjective state of symptoms preoperatively and at a 3-month follow-up after the treatment, the earlier study relied on patient diaries collected retrospectively. In the second study, Licow et al. performed an ongoing prospective examination of 50 women with OAB, the results of which they published in a small preliminary report [21]. They found a significant improvement in symptoms of OAB as well as improved quality of life. However, they included unselected phenotypes of OAB and failed to report the number of non-responders or the time elapsed until retreatment. Altogether, unlike previous women-only studies, our study was the first prospective one, included only women with iOAB, and used internationally validated questionnaires to reliably demonstrate the effects of treatment with 100 U onabotulinumtoxin A.

The characteristics of patients in our study were mostly in line with those reported in previous studies, except for the higher mean age of our patients. In our study, the mean age of patients was 68 years, whereas in previous studies it ranged from 56 to 63 years [2, 28]. As aging is a well-known risk factor for OAB, the excellent efficacy of 100-U onabotulinumtoxin A treatment as revealed in our study is somehow surprising. More specifically, the efficacy of treatment with 100 U onabotulinumtoxin A was shown to be similar among women in older and younger subgroups. Surprisingly, we also observed that the efficacy of the treatment was even greater among women with overweight than among those with normal weight. Considering these results, we can assume that onabotulinumtoxin A treatment is an effective treatment for elderly and overweight women with iOAB without a higher risk of retention. In terms of the characteristics between the good and poor outcome groups, the only significant difference concerned diagnoses of neurological diseases. In the poor outcome group, all three patients had previously been diagnosed with a neurological disease, whereas in the good outcome group the prevalence of neurological disease was 33%. Indeed, although the aim of this study was to focus on iOAB, we found that all three patients with a poor outcome had a neurological diagnose code. Thus, it may be possible that these patient, after all, may have had an underlying neurological factor of OAB. Although this possibility should not notably affect our conclusions because the size of this group was so small, an underestimation of the effectiveness of onabotulinumtoxin A treatment may have occurred. Altogether, to the best of our knowledge, our study was the first to show the number and characteristics of non-responders.

The clinical effectiveness of onabotulinumtoxin A treatment seems to be the result of many factors. Onabotulinumtoxin A decreases urinary urgency by relaxing the bladder, but also by decreasing the number of sensory receptors in the bladder [16, 28, 29]. After onabotulinumtoxin A treatment, the detrusor muscle relaxes and afferent signaling of the bladder is dampened, resulting in the relief of symptoms [8, 30] and allowing patients to learn new urinary habits and modify bladder function through behavioral therapy. This in turn helps these patients to better tolerate the feeling of a full bladder, even after the effects of onabotulinumtoxin A have faded. Thus, it appears that those studies that included patients with unselected phenotypes of OAB may have underestimated the effect of the 100-U dose for iOAB. Furthermore, the time interval until symptoms reappear, and retreatment is needed, may vary depending on the type of OAB. Altogether, the etiology of the iOAB is still partly unknown and likely to be a sum of many different mechanisms, as the recent studies on the connection between vaginal and urine microbiome and the intensity of the symptoms of OAB has been shown [31]. Considering this etiological diversity of iOAB, new treatment methods such as intervention of the urine microbiome and vaginal and bladder mucosal laser treatments are currently under research and may become second-line treatment alongside onabotulinumtoxin A in the future [32, 33].

Previous studies have shown that the most important AEs of onabotulinumtoxin A treatment are UTI and urinary retention, which requires temporary CIC. The incidence of AEs increases as the dose of onabotulinumtoxin A increases. At a dose of 300 U, for example, 21.1–57% of patients experience a UTI after treatment, and 16.4–88.2% of patients require CIC after treatment [11]. Comparing previous studies on 100-U onabotulinumtoxin A treatment, Licow et al. used fosfomycin as a prophylactic antibiotic for 3 consecutive days, starting on the day before treatment, with the results revealing the lowest rate of AEs: with 4% of patients experiencing infections and 2% requiring CIC [21]. Meanwhile, Chapple et al. and Abreu-Mendes et al., neither of whom used prophylactic antibiotics, generated results showing more UTIs, with percentages of 20.4% and 19.9% respectively [1, 15]. In our study, the prophylactic ciprofloxacin was used, and the resulting total AE rate was 17.0%: 9.6% of patients had a UTI and 6.4% required short-term CIC, both reasonable percentages in comparison with those reported in previous studies. Furthermore, we could not find any specific patient characteristics that increased the risk of retention. Although the use of prophylactic antibiotics did help to prevent infections, it is advisable to use the lowest effective dose of onabotulinumtoxin A as the retention caused by the treatment increases the risk of infections owing to weaker bladder emptying. Overall, the risk of AEs from the treatment of iOAB with 100 U of onabotulinumtoxin A seems to be acceptably low in all types of patients.

Our study had several strengths but also some limitations. First, pursuing a prospective follow-up study among members of the Finnish population provided a good starting point owing to the equal availability of public health care among the population and its minimal ethnic variation. Second, unlike most previous studies, we focused solely on women with iOAB; men and neurogenic OAB were excluded to minimize the heterogeneity of the study population. Furthermore, we standardized the dose of onabotulinumtoxin A to 100 U. All patients included in the study had first undergone first-line treatment according to current Finnish care guidelines [34], including those pertaining to medication use. Onabotulinumtoxin A treatments were performed in one hospital by two specialized urogynecologists, with the injection locations and numbers in the bladder standardized. We were also able to compare the effectiveness of onabotulinumtoxin A treatment based on age and BMI, unlike in earlier studies, and were additionally able to show similar results among older and better results among overweight women. Regarding the limitations of our study, we did not perform a power calculation and the study group was relatively small; thus, the number of patients exhibiting poor outcomes was low: only three patients. Owing to this small number, we were unable to comprehensively determine any specific characteristic that could reliably predict unsatisfactory outcomes of onabotulinumtoxin A treatment for iOAB. The response rate of 78% was low for prospective study and it may have an impact on the reliability of the results. Furthermore, we did not perform a preoperative urodynamic examination on patients to show detrusor overactivity. Although all patients were instructed to contact our clinic in the event that they experienced any AEs from treatment, some patients may have contacted another health care unit. As such, the number of patients who experienced AEs may have been slightly underestimated. Lastly, even though the mean time interval between the first and second treatment was 15 months, the range was exceedingly wide, from 4 to 66 months, and the follow-up time for the last treated patients was only 3 months. Thus, because of this limitation, we were unable to reliably demonstrate the time that elapsed between necessary retreatments. Furthermore, a large number of patients did not contact us after their first treatment, and we therefore lack data on the reasons why they did not seek retreatment. It is possible, however, that some of these patients may have received permanent relief from their symptoms after only a single onabotulinumtoxin A treatment. Because of these limitations in our study, we recommend that studies on the effectiveness of onabotulinumtoxin A and the duration of the effect in a larger study population are carried out in the future.

## Conclusion

Our study showed that 100-U onabotulinumtoxin A is a safe and very effective second-line treatment of iOAB in women. The rate of AEs that occur at a 100-U dose of onabotulinumtoxin A is quite low, whereas the beneficial effects of the treatment persist for a relatively long time, even after a single treatment.

## Data Availability

The data that support the findings of this study are not openly available due to reasons of sensitivity and are available from the corresponding author Henna Rossi upon reasonable request.

## Abbreviations

AE: Adverse effect
CIC: Clean intermittent catheterization
IIQ-7: Incontinence Impact Questionnaire
iOAB: Idiopathic overactive bladder
OAB: Overactive bladder
PGI-I: Patient Global Index of Improvement
UDI-6: Urogenital Distress Inventory
UTI: Urinary tract infection
VAS: Visual Analogue Scale
